# Prevalence and death rate of birth defects from population-based surveillance in Hunan Province, China, 2010–2020

**DOI:** 10.1038/s41598-024-65072-7

**Published:** 2024-06-25

**Authors:** Xu Zhou, Donghua Xie, Yurong Jiang, Junqun Fang

**Affiliations:** Hunan Provincial Maternal and Child Health Care Hospital, Changsha, 410000 Hunan Province China

**Keywords:** Medical research, Epidemiology

## Abstract

To describe the prevalence and death rate of birth defects from population-based surveillance in Hunan Province, China. Data were obtained from the population-based Birth Defects Surveillance System in Hunan Province, China (2010–2020). The surveillance population included all live births, stillbirths, infant deaths, and legal terminations of pregnancy from 28 weeks of gestation to 42 days after birth between 2010 and 2020 when the mother resided in the surveillance area (Liuyang County and Shifeng District, Hunan Province). The prevalence of birth defects is the number of birth defects per 1000 infants (‰). The death rate of birth defects is the number of deaths attributable to birth defects per 100 birth defects (%). The prevalence and death rate with 95% confidence intervals (CI) were calculated by the log-binomial method. Crude odds ratios (ORs) were calculated to examine the association of each demographic characteristic with birth defects. Our study included 228,444 infants, and 4453 birth defects were identified, with a prevalence of 19.49‰ (95%CI 18.92–20.07). Congenital heart defects were the most common specific defects (5.29‰), followed by limb defects (4.01‰). Birth defects were more common in males than females (22.34‰ vs. 16.26‰, OR = 1.38, 95%CI 1.30–1.47), in premature birth than not (91.82‰ vs. 16.14‰, OR = 6.16, 95%CI 5.72–6.65), in birth weight < 2500 g (98.26‰ vs. 16.22‰, OR = 6.61, 95%CI 6.11–7.15) or > 4000 g (19.48‰ vs. 16.22‰, OR = 1.21, 95%CI 1.03–1.42) than birth weight 2500–4000 g, in hospitalized deliveries than other institutions (22.16‰ vs. 11.74‰, OR = 1.91, 95%CI 1.76–2.07), in multiple births than singletons (28.50‰ vs. 19.28‰, OR = 1.49, 95%CI 1.27–1.76), in maternal age < 20 years (26.33‰ vs. 18.69‰, OR = 1.42, 95%CI 1.15–1.76) or >  = 35 years (24.31‰ vs. 18.69‰, OR = 1.31, 95%CI 1.18–1.45) than maternal age 25–29 years, and in number of pregnancies >  = 4 (22.91‰ vs. 18.92‰, OR = 1.22, 95%CI 1.10–1.35) than the first pregnancy. A total of 747 deaths attributable to birth defects were identified, including 603 (80.72%) stillbirths, 75 (10.04%) deaths within 7 days after birth, 46 (6.16%) deaths in 7–27 days after birth, 23 (3.08%) deaths in 28–42 days after birth. The death rate of birth defects was 16.78% (95%CI 15.57–17.98). Deaths attributable to birth defects accounted for 51.09% (747/1462) of all deaths. Central nervous system defects had the highest death rate (90.27%), and neonatal genetic metabolic defects had the lowest death rate (0.39%). In summary, we have described the prevalence and epidemiology of birth defects from population-based surveillance in Hunan Province, China, 2010–2020. There were differences in the prevalence and death rate of birth defects between population-based surveillance and hospital-based surveillance.

## Introduction

Birth defects are structural or functional anomalies at or before birth^1^. The observed prevalence of birth defects is 2–3% worldwide^2^ and is estimated to be 4–6% in China^3^. Birth defects are associated with perinatal deaths^4–7^. In developed countries such as Europe and the United States, birth defects have been the leading cause of perinatal death and infant death for a long time^8^. World Health Organization (WHO) estimated that about 12.6% of neonatal deaths globally are related to birth defects each year^9^. About 240,000 newborns worldwide die from birth defects within the first 28 days of life, and birth defects cause 170,000 children's deaths between the ages of 1 month and 5 years each year^10^. Therefore, research on birth defects and deaths attributable to birth defects is significant and deserves more attention.

There were two main methods of birth defect surveillance in the world: hospital-based and population-based. Population-based surveillance is the method recommended by the WHO^11^. Population-based surveillance has a defined source population (typically defined by maternal residence). All identified congenital anomalies occurring within that source population are ascertained and included, regardless of the delivery site. In contrast, the source population for hospital-based surveillance typically cannot be defined accurately. Hospital-based surveillance usually covers at least a few hospitals or clinics in one geographic region. However, there generally are no distinct catchment areas for specific hospitals and thus no defined denominator of the entire source population from which all cases are ascertained^11^. Therefore, population-based surveillance is better suited to describing the prevalence of birth defects than hospital-based surveillance. However, due to a lack of resources or other restrictions, some countries may find it challenging to conduct a population-based surveillance program and may choose to begin with the development of a facility- or hospital-based program. For example, in developed countries such as the United States and Europe, population-based surveillance is the primary method^12,13^, whereas, in developing countries such as Latin America and China, hospital-based surveillance is the primary method^3,14^.

To our knowledge, there are fewer population-based surveillance studies of birth defects in China. The following are exist studies based on population-based surveillance: Zhang et al.^15^ compared the prevalence of birth defects between assisted reproductive technology-conceived offspring and spontaneously-conceived offspring in Beijing, 2014–2015; Zhou et al.^16^ described the epidemiology of birth defects in Southern Jiangsu (2014–2018); Jiang et al.^17^ evaluated the effect of preconception examinations programs on the prevention of birth defects in Dongguan City, Guangdong Province (2013–2017); Xiong et al.^18^ compared the epidemiology of birth defects between the rural and urban areas in Hunan Province (2014–2018); Lin et al.^19^ described the epidemiology and environmental risk factors of birth defects in Liuyang City, Hunan Province (2013–2014); Xiong et al.^20^ evaluated the relationship between air pollution and birth defects in Hunan Province (2014–2016); Xie et al.^21^ evaluated the perinatal outcomes and prognosis of congenital heart defects in Hunan Province (2010–2012).

However, these studies have limitations. First, as mentioned above, there are few population-based studies, especially on the prevalence and epidemiology of birth defects in China. Our study may make some original contributions to the field. Second, compared with hospital-based surveillance, population-based surveillance may provide some additional indicators for birth defects, such as the prevalence of birth defects by gestational age at termination of pregnancy, birth weight, delivery site, number of fetuses, number of pregnancies, and parities, which cannot be reported in hospital-based surveillance in China^3^. Third, some previous studies had limited data, such as relatively few cases included, or surveys were conducted in unrepresentative districts or for a short period. Fourth, some studies needed to be updated.

Therefore, we conducted a comprehensive analysis of population-based surveillance in Hunan Province, China, from 2010–2020 to describe the prevalence and death rate of birth defects, which may make some original contributions to the field.

## Methods

### Data sources

The data was obtained from the population-based Birth Defects Surveillance System in Hunan Province, China, run by the Hunan Provincial Health Commission. In 2008, the Hunan Provincial Health Commission selected Liuyang County and Shifeng District as surveillance sites, and experts comprehensively evaluated the two sites before this decision was made. The two sites are located in the central Hunan Province, with about 1.5–2 million permanent residents and 20,000 live births per year, and their geographic location, demographics, economic conditions, and healthcare facilities can mirror the entire province.

The surveillance population was infants (or offsprings) whose mothers lived in Liuyang County and Shifeng District between 2010 and 2020, including all live births, stillbirths, infant deaths, and legal termination of pregnancy from 28 weeks of gestation to 42 days after birth. Surveillance data included demographic characteristics such as sex, residence, gestational age at termination of pregnancy, birth weight, delivery site, number of fetuses, maternal age, number of pregnancies, and parities.

The maternal and child healthcare workers at the community health service centers in urban areas and village doctors in rural areas are responsible for collecting surveillance data. They will follow up on all infants until 42 days after birth and use a standardized form to collect surveillance data. According to the "Maternal and Child Health Monitoring Manual in Hunan Province" formulated by the Hunan Provincial Health Commission, diagnostic methods for birth defects include clinical examination, ultrasound examination, biochemical examination, chromosomal analysis, genetic testing, autopsy, and other appropriate examinations. All birth defects should be diagnosed by medical institutions at district and county levels and above as soon as possible. Each quarter, doctors at county-level surveillance centers collect and review the surveillance data from the maternal and child healthcare workers at the community health service centers in urban areas and village doctors in rural areas and then submit it to the municipal surveillance centers for review. After the municipal surveillance centers finish the review, they will submit it to the provincial surveillance center (the Hunan Provincial Maternal and Child Health Care Hospital) for review.

Birth defects are coded according to the WHO International Classification of Diseases (Tenth Revision, ICD-10). The ICD codes of specific defects are shown in Table 2.

### Ethics approval and consent to participate

The Medical Ethics Committee of Hunan Provincial Maternal and Child Health Care Hospital approved the study. (NO: 2023-S015). It is a retrospective study of medical records; all data were fully anonymized before we accessed them. Moreover, we de-identified the patient records before analysis. We confirmed that all operations followed relevant guidelines and regulations. We confirmed that informed consent was obtained from all subjects or their legal guardian(s). Data collectors (maternal and child healthcare workers and village doctors) obtain consent from infants' parents or guardians before collecting surveillance data, and infants' families witness it. Since the Health Commission of Hunan Province collects those data, and the government has emphasized the privacy policy in the "Maternal and Child Health Monitoring Manual in Hunan Province" and has a strict work management system, no additional written informed consent is needed.

### Data quality control

To carry out surveillance, the Hunan Provincial Health Commission formulated the "Maternal and Child Health Monitoring Manual in Hunan Province", and all levels of government support this work. Data were collected and reported by experienced data collectors. All data collectors must be trained and qualified before starting work. The Hunan Provincial Health Commission asked the technical guidance departments to conduct comprehensive quality control yearly to reduce surveillance data integrity and information error rates. To ensure that all cases of birth defects are accurately diagnosed and classified, all cases are reviewed by provincial doctors.

### Definitions

Prevalence of birth defects is the number of birth defects per 1000 infants (‰). The death rate of total infants is the number of deaths per 100 infants (%). The death rate of birth defects is the number of deaths attributable to birth defects per 100 birth defects (%).

### Statistical analysis

The prevalence and death rate of birth defects and its 95% confidence interval (CI) were calculated by the log-binomial method. Crude odds ratios (ORs) were calculated to examine the association of each demographic characteristic with birth defects.

Statistical analyses were performed using SPSS 18.0 (IBM Corp., NY, USA).

## Results

### Prevalence of birth defects

Our study included 228,444 infants, and 4453 birth defects were identified, with a prevalence of 19.49‰ (95%CI 18.92–20.07). From 2010 to 2020, the prevalences of birth defects were 17.34‰, 18.98‰, 20.09‰, 12.69‰, 25.74‰, 29.29‰, 14.73‰, 21.76‰, 18.45‰, 16.03‰, and 16.18‰, respectively. The highest prevalence of birth defects was in 2015 (29.29‰), and the lowest was in 2013 (12.69‰). (Table 1).

**Table 1:** Prevalence of birth defects in Hunan Province, China, 2010–2020.

Year	Infants (n)	Birth defects (n)	Prevalence (‰, 95%CI)
2010	19,260	334	17.34(15.48–19.20)
2011	20,284	385	18.98(17.08–20.88)
2012	22,850	459	20.09(18.25–21.93)
2013	22,932	291	12.69(11.23–14.15)
2014	22,299	574	25.74(23.64–27.85)
2015	23,284	682	29.29(27.09–31.49)
2016	22,943	338	14.73(13.16–16.30)
2017	24,863	541	21.76(19.93–23.59)
2018	20,654	381	18.45(16.59–20.30)
2019	16,035	257	16.03(14.07–17.99)
2020	13,040	211	16.18(14.00–18.36)
Total	228,444	4453	19.49(18.92–20.07)

*CI* Confidence intervals.

### Prevalence of specific defects

The prevalences of congenital heart defects, limb defects, neonatal genetic metabolic defects, ear defects, urogenital defects, cleft lip and/or palate, central nervous system defects, digestive system defects, and chromosomal abnormalities were 5.29‰, 4.01‰, 3.41‰, 1.91‰, 1.09‰, 0.84‰, 0.49‰, 0.46‰, and 0.42‰, respectively. Congenital heart defects were the most common specific defects (5.29‰), followed by limb defects (4.01‰). Table 2 shows the details of the prevalences of specific defects. (Table 2).

**Table 2:** Prevalence of specific defects.

Types of birth defects	ICD codes	Birth defects (n) (N: 228,444)	Prevalence (‰, 95%CI)
Congenital heart defects	Q20–Q26	1208	5.29(4.99–5.59)
Limb defects	Q69–Q74	917	4.01(3.75–4.27)
Polydactyly	Q69	472	2.07(1.88–2.25)
Clubfoot	Q66.0	227	0.99(0.86–1.12)
Syndactyly	Q70	108	0.47(0.38–0.56)
Limb reduction	Q71–Q73	52	0.23(0.17–0.29)
Other limb defects	Other codes, excluding the above	77	0.34(0.26–0.41)
Neonatal genetic metabolic defects	E03, E25.0, E70–E90, D55.0	779	3.41(3.17–3.65)
Glucose-6-phosphate dehydrogenase	D55.0	677	2.96(2.74–3.19)
Congenital hypothyroidism	E03	101	0.44(0.36–0.53)
Phenylketonuria	E70.0	1	0.00(0.00–0.02)
Ear defects	Q16–Q17	437	1.91(1.73–2.09)
Anotia/microtia	Q17.2, Q16.0	79	0.35(0.27–0.42)
Other external ear defects	Other codes, excluding the above	368	1.61(1.45–1.78)
Urogenital defects	Q54, Q60–Q64	250	1.09(0.96–1.23)
Kidney defects	Q60	146	0.64(0.54–0.74)
Hypospadias	Q54	101	0.44(0.36–0.53)
Other urogenital defects	Other codes, excluding the above	3	0.01(0.00–0.04)
Cleft lip and/or palate	Q35–Q37	193	0.84(0.73–0.96)
Cleft lip with palate	Q37	72	0.32(0.24–0.39)
Cleft palate	Q35	72	0.32(0.24–0.39)
Cleft lip	Q36	49	0.21(0.16–0.28)
Central nervous system defects	Q00–Q07	113	0.49(0.40–0.59)
Hydrocephalus	Q03	61	0.27(0.20–0.33)
Spina bifida	Q05	19	0.08(0.05–0.13)
Other central nervous system defects	Other codes, excluding the above	36	0.16(0.11–0.22)
Digestive system defects	Q38–Q45	105	0.46(0.37–0.55)
Atresia of rectum and anus	Q42	52	0.23(0.17–0.29)
Esophageal atresia	Q39	11	0.05(0.02–0.09)
Other digestive system defects	Other codes, excluding the above	42	0.18(0.13–0.25)
Chromosomal abnormalities	Q90–Q99	96	0.42(0.34–0.50)
Down syndrome	Q90	48	0.21(0.15–0.28)
Other chromosomal abnormalities (Aneuploid chromosomes or large chromosome deletions)	Other codes, excluding the above	48	0.21(0.15–0.28)
Other defects	Other codes of Q00–Q99	520	2.28(2.08–2.47)

Some cases with multiple specific defects.

*N* Number of infants; *CI* Confidence intervals; *ICD* International Classification of Diseases.

### Prevalence of birth defects by demographic characteristics

Birth defects were more common in males than females (22.34‰ vs. 16.26‰, OR = 1.38, 95%CI 1.30–1.47), in premature birth than not (91.82‰ vs. 16.14‰, OR = 6.16, 95%CI 5.72–6.65), in birth weight < 2500 g (98.26‰ vs. 16.22‰, OR = 6.61, 95%CI 6.11–7.15) or > 4000 g (19.48‰ vs. 16.22‰, OR = 1.21, 95%CI 1.03–1.42) than birth weight 2500–4000 g, in hospitalized deliveries than other institutions (22.16‰ vs. 11.74‰, OR = 1.91, 95%CI 1.76–2.07), in multiple births than singletons (28.50‰ vs. 19.28‰, OR = 1.49, 95%CI 1.27–1.76), in maternal age < 20 years (26.33‰ vs. 18.69‰, OR = 1.42, 95%CI 1.15–1.76) or >  = 35 years (24.31‰ vs. 18.69‰, OR = 1.31, 95%CI 1.18–1.45) than maternal age 25–29 years, and in number of pregnancies >  = 4 (22.91‰ vs. 18.92‰, OR = 1.22, 95%CI 1.10–1.35) than the first pregnancy. There was no significant difference in the prevalence of birth defects between urban and rural areas (OR = 0.98, 95%CI 0.92–1.04) or between different parities (OR values contain 1) (Table 3).

**Table 3:** Prevalence of birth defect by demographic characteristics.

Indicator	Infants (n)	Birth defects (n)	Prevalence (‰, 95%CI)	OR(95%CI)
Sex				
Male	120,436	2691	22.34(21.50–23.19)	1.38(1.30–1.47)
Female	108,000	1756	16.26(15.50–17.02)	Reference
Unknown	8	6	–	–
Residence				
Urban	74,883	1437	19.19(18.20,20.18)	0.98(0.92–1.04)
Rural	153,561	3016	19.64(18.94,20.34)	Reference
Premature birth (gestational age at termination of pregnancy)				
No (> = 37 weeks)	218,315	3523	16.14(15.60–16.67)	Reference
Yes (< 37 weeks)	10,129	930	91.82(85.91–97.72)	6.16(5.72–6.65)
Birth weight (g)				
2500–4000	211,489	3430	16.22(15.68–16.76)	Reference
< 2500	8793	864	98.26(91.71–104.81)	6.61(6.11–7.15)
> 4000	8162	159	19.48(16.45–22.51)	1.21(1.03–1.42)
Delivery site				
Hospital (above the district or county level)	169,933	3766	22.16(21.45–22.87)	1.91(1.76–2.07)
Other institutions (township health center or other)	58,511	687	11.74(10.86–12.62)	Reference
Number of fetuses				
Singletons	223,110	4301	19.28(18.70–19.85)	Reference
Multiple births	5334	152	28.50(23.97–33.03)	1.49(1.27–1.76)
Maternal age (years old)				
< 20	3418	90	26.33(20.89–31.77)	1.42(1.15–1.76)
20–24	58,310	1154	19.79(18.65–20.93)	1.06(0.98–1.14)
25–29	97,167	1816	18.69(17.83–19.55)	Reference
30–34	49,518	906	18.30(17.10–19.49)	0.98(0.90–1.06)
> = 35	20,031	487	24.31(22.15–26.47)	1.31(1.18–1.45)
Number of pregnancies				
1 (First pregnancy)	93,183	1763	18.92(18.04–19.80)	Reference
2	86,182	1627	18.88(17.96–19.80)	1.00(0.93–1.07)
3	29,178	607	20.80(19.15–22.46)	1.10(1.00–1.21)
> = 4	19,901	456	22.91(20.81–25.02)	1.22(1.10–1.35)
Parities				
1 (First delivery)	117,163	2281	19.47(18.67–20.27)	Reference
2	102,822	1983	19.29(18.44–20.13)	0.99(0.93–1.05)
> = 3	8459	189	22.34(19.16–25.53)	1.15(0.99–1.34)

*CI* Confidence intervals; *OR* crude odds ratio.

### Death rate of birth defects

A total of 1462 deaths were identified, including 1255 (85.84%) stillbirths, 134 (9.17%) deaths within 7 days after birth, 50 (3.42%) deaths between 7 and 27 days after birth, and 23 (1.57%) deaths between 28 and 42 days after birth. A total of 747 deaths attributable to birth defects were identified, including 603 (80.72%) stillbirths, 75 (10.04%) deaths within 7 days after birth, 46 (6.16%) deaths between 7 and 27 days after birth, 23 (3.08%) deaths between 28 and 42 days after birth. Deaths attributable to birth defects accounted for 51.09% (747/1462) of total deaths. The total death rate was 0.64% (95%CI 0.61–0.67), and the death rate of birth defects was 16.78% (95%CI 15.57–17.98), which was significantly higher than the total death rate (OR = 31.29, 95%CI 28.49–34.38).

The death rates of congenital heart defects, limb defects, neonatal genetic metabolic defects, ear defects, urogenital defects, cleft lip and/or palate, central nervous system defects, digestive system defects, and chromosomal abnormalities were 23.43%, 8.40%, 0.39%, 1.60%, 40.80%, 28.50%, 90.27%, 41.90%, and 52.08%, respectively. Central nervous system defects had the highest death rate (90.27%), and neonatal genetic metabolic defects had the lowest death rate (0.39%). Table 4 shows the details of the death rate of specific defects. (Table 4).

**Table 4:** Death rate of birth defects.

Types	n	Total deaths (n)	Death rate (%, 95%CI)	Stillbirths		Deaths within 7 days after birth		Deaths between 7 and 27 days after birth		Deaths between 28 and 42 days after birth	
				n	Proportion (%)	n	Proportion (%)	n	Proportion (%)	n	Proportion (%)
Total infants	228,444	1462	0.64(0.61–0.67)	1255	85.84	134	9.17	50	3.42	23	1.57
Total birth defects	4453	747	16.78(15.57–17.98)	603	80.72	75	10.04	46	6.16	23	3.08
Congenital heart defects	1208	283	23.43(20.70–26.16)	211	74.56	31	10.95	29	10.25	12	4.24
Limb defects	917	77	8.40(6.52–10.27)	60	77.92	10	12.99	2	2.60	5	6.49
Polydactyly	472	11	2.33(0.95–3.71)	7	63.64	3	27.27	0	0.00	1	9.09
Clubfoot	227	30	13.22(8.49–17.95)	28	93.33	1	3.33	0	0.00	1	3.33
Syndactyly	108	8	7.41(2.27–12.54)	4	50.00	3	37.50	1	12.50	0	0.00
Limb reduction	52	24	46.15(27.69–64.62)	20	83.33	1	4.17	1	4.17	2	8.33
Other limb defects	77	7	9.09(2.36–15.83)	4	57.14	2	28.57	0	0.00	1	14.29
Neonatal genetic metabolic defects	779	3	0.39(− 0.05–0.82)	0	0.00	3	100.00	0	0.00	0	0.00
Glucose-6-phosphate dehydrogenase	677	3	0.44(− 0.06–0.94)	0	0.00	3	100.00	0	0.00	0	0.00
Congenital hypothyroidism	101	0	0.00(0.00–0.00)	0	–	0	–	0	–	0	–
Phenylketonuria	1	0	0.00(0.00–0.00)	0	–	0	–	0	–	0	–
Ear defects	437	7	1.60(0.42–2.79)	3	42.86	4	57.14	0	0.00	0	0.00
Anotia/microtia	79	3	3.80(− 0.50–8.09)	1	33.33	2	66.67	0	0.00	0	0.00
Other external ear defects	368	4	1.09(0.02–2.15)	2	50.00	2	50.00	0	0.00	0	0.00
Urogenital defects	250	102	40.80(32.88–48.72)	95	93.14	5	4.90	1	0.98	1	0.98
Kidney defects	146	90	61.64(48.91–74.38)	86	95.56	4	4.44	0	0.00	0	0.00
Hypospadias	101	10	9.90(3.76–16.04)	7	70.00	1	10.00	1	10.00	1	10.00
Other urogenital defects	3	2	66.67(− 25.73–159.06)	2	100.00	0	0.00	0	0.00	0	0.00
Cleft lip and/or palate	193	55	28.50(20.97–36.03)	50	90.91	3	5.45	2	3.64	0	0.00
Cleft lip with palate	72	43	59.72(41.87–77.57)	41	95.35	2	4.65	0	0.00	0	0.00
Cleft palate	72	3	4.17(− 0.55–8.88)	0	0.00	1	33.33	2	66.67	0	0.00
Cleft lip	49	9	18.37(6.37–30.37)	9	100.00	0	0.00	0	0.00	0	0.00
Central nervous system defects	113	102	90.27(72.75–107.78)	92	90.20	6	5.88	2	1.96	2	1.96
Hydrocephalus	61	56	91.80(67.76–115.85)	53	94.64	2	3.57	1	1.79	0	0.00
Spina bifida	19	16	84.21(42.95–125.47)	13	81.25	2	12.50	0	0.00	1	6.25
Other central nervous system defects	36	33	91.67(60.39–122.94)	28	84.85	3	9.09	1	3.03	1	3.03
Digestive system defects	105	44	41.90(29.52–54.29)	25	56.82	7	15.91	8	18.18	4	9.09
Atresia of rectum and anus	52	13	25.00(11.41–38.59)	6	46.15	3	23.08	3	23.08	1	7.69
Esophageal atresia	11	10	90.91(34.56–147.26)	3	30.00	1	10.00	5	50.00	1	10.00
Other digestive system defects	42	21	50.00(28.61–71.39)	16	76.19	3	14.29	0	0.00	2	9.52
Chromosomal abnormalities	96	50	52.08(37.65–66.52)	39	78.00	2	4.00	8	16.00	1	2.00
Down syndrome	48	22	45.83(26.68–64.99)	14	63.64	0	0.00	7	31.82	1	4.55
Other chromosomal abnormalities (Aneuploid chromosomes or large chromosome deletions)	48	28	58.33(36.73–79.94)	25	89.29	2	7.14	1	3.57	0	0.00
Other defects	520	96	18.46(14.77–22.15)	76	79.17	15	15.63	3	3.13	2	2.08

*CI* Confidence intervals.

## Discussion

Overall, we have described the epidemiology of birth defects. Our study is the latest systematic study on the epidemiology of birth defects based on population-based surveillance in Hunan Province, China, which makes some original contributions to the field.

There are several interesting findings from this study. First, the overall prevalence of birth defects was 19.49‰, consistent with the observed global prevalence of birth defects (about 2%–3%)^2^. The following prevalences of birth defects were reported in several countries: 23.9‰ in Europe (2003–2007)^22^, 298.6 per 10,000 pregnancies in Japan (2011–2014)^23^, 44.63‰ in Korea (2008–2014)^24^, 1.1% of malformed newborns in the Latin American network for congenital malformation surveillance (2017–2019)^25^, 6.62‰ in Uganda (2015–2017)^26^, and 18.45‰ in India (2018)^27^. The prevalence of birth defects in China is estimated to be 4–6%^3^, which is higher than the prevalence in this study. In addition, the reported prevalence of birth defects in some regions of China is significantly lower than the estimated prevalence. The following prevalences of birth defects were obtained from population-based surveillance: 15.55‰ in southern Jiangsu (2014–2018)^16^, 13.46‰ in Dongguan City, Guangdong Province (2013–2017)^17^, 22.05‰ in Hunan Province (2014–2018)^18^; the following prevalences were obtained from hospital-based surveillance: 18.89‰ in Hunan Province (2010–2020)^28^, 13.55‰ in Guilin, Guangxi Zhuang Autonomous Region (2018–2020)^29^, and 7.15‰ in Southern Jiangsu Province (2014–2018)^30^. Such considerable differences in prevalence may be mainly related to the diagnosis and reporting rates of birth defects, as many minor defects are not reported, and many defects are difficult to diagnose^31^. There were also significant differences in prevalence between population-based and hospital-based surveillance. It may be mainly related to differences in the surveillance population and periods, as hospital-based surveillance mainly includes fetuses between 28 weeks of gestation and 7 days after birth in hospitals, while population-based surveillance includes fetuses between 28 weeks gestation and 42 days after birth in given district or region^3,32^.

The prevalences of many specific defects were significantly lower than those obtained from hospital-based surveillance, such as congenital heart defects, limb reduction, hypospadias, hydrocephalus, and esophageal atresia. In contrast, the prevalence of several specific defects was significantly higher than the prevalence obtained from hospital-based surveillance, such as Down syndrome (Zhou et al.^28^, Hunan Province, China, 2010–2020). It may be mainly related to the selection of hospital-based surveillance sites and the surveillance population. On the one hand, in the hospital-based surveillance program, the government prefers to select the central delivery institutions (most have good medical services) in given district or region as hospital surveillance sites, which may also be the first choice for pregnant women with abnormal pregnancies (including birth defects), resulting in a high prevalence of some specific defects; On the other hand, the leading delivery institutions have well-established prenatal screening and diagnostic services, and several specific defects (such as Down syndrome) are diagnosed and terminated before 28 weeks, resulting in a low prevalence^33^. It is also the reason for the prevalence of some specific defects significantly lower than the globally reported prevalence, such as Down syndrome (almost 1 in 600 live births) , urogenital defects (3–6 out of 1.000 of newborns) , hydrocephalus (67.5–316.1 per 100,000 births)^34^, spina bifida (36.08–243.14 per 100,000 fetuses)^35^. In addition, the prevalence of neonatal genetic metabolic defects was relatively high in this study, while some regions hardly report genetic metabolic disorders (such as glucose-6-phosphate dehydrogenase) as birth defects.

Second, there was no significant difference in the prevalence of birth defects between urban and rural areas in this study. However, in many studies based on hospital-based surveillance, birth defects were more common in urban areas than in rural areas^28,36^. It may be mainly associated with economic and medical conditions. In the hospital-based surveillance program, urban areas are associated with better economic and medical conditions, which may increase birth defect diagnosis and reporting rates^37^. In the population-based surveillance program, surveillance data are obtained through follow-up surveys, and most birth defects can be detected and registered even if they are not diagnosed before birth or within 7 days of after birth. Our findings suggested that population-based surveillance programs can avoid the biases between urban and rural areas.

Third, based on detailed surveillance data, we have presented the prevalence of birth defects by gestational age at termination of pregnancy, birth weight, delivery site, number of fetuses, number of pregnancies, and parity, which cannot be reported in hospital-based surveillance in China^3^. In addition, we found that birth defects were more common in premature birth, birth weight < 2500 g or > 4000 g, hospitalized deliveries, multiple births, and number of pregnancies >  = 4. Although similar findings have been reported in the past^38,39^, our study provided detailed risk values (OR), and our study was based on long-term systematic surveillance data, which may be helpful in clinical counseling.

Fourth, birth defects significantly increase the risk of death. In this study, the death rate of birth defects was 16.78%, which was lower than the perinatal mortality rate (from 28 weeks of gestation to 7 days after birth) of birth defects (25.03%) in Hunan Province, China (hospital-based surveillance, 2010–2020)^40^. It may be mainly associated with differences in the surveillance population. As discussed above, pregnant women with abnormal pregnancies (including birth defects) are more likely to go to hospital-based surveillance sites, which may result in more deaths attributable to birth defects in hospital-based surveillance than in population-based surveillance.

Significant differences in the death rates of specific defects reflected the severity of specific defects. Overall, the death rate was higher in infants with specific defects that may significantly affect the physiological functions (such as central nervous system defects and chromosomal abnormalities) or appearance (such as cleft lip and/or palate). To the best of our knowledge, there are fewer studies on deaths attributable to birth defects and fewer studies available for comparison^40–45^. Compared to the perinatal mortality rate of birth defects in Hunan Province, China (hospital-based surveillance, 2010–2020), death rates were higher for several specific defects, such as limb reduction (46.15% vs. 35.83%), spina bifida (84.21% vs. 63.64%), and esophageal atresia (90.91% vs. 32.41%); In contrast, death rates were lower for several specific defects, such as Down syndrome (45.83% vs. 59.09%)^40^. On the one hand, many specific defects are difficult to diagnose before birth, and many infants with defects die after the first 7 days of life. For example, in this study, 60% of deaths attributable to esophageal atresia, 18.75% of deaths attributable to spina bifida, and 12.5% of deaths attributable to limb reduction occurred after the first 7 days of life. It is the main reason why death rates of limb reduction, spina bifida, and esophageal atresia were higher than the perinatal mortality rate (hospital-based surveillance). On the other hand, with advances in medical conditions (including prenatal screening and diagnostic services), more and more birth defects are diagnosed and terminated, and these services are more easily provided to pregnant women in hospital-based surveillance systems^33^. It is the main reason why the death rate of Down syndrome was lower than the perinatal mortality rate (hospital-based surveillance).

Some things could be improved in our study. First, we could not merge the case information due to electronic upgrades to the Birth Defects Surveillance System in 2013 and 2015. It prevented us from conducting a multifactorial analysis. Second, specific defects, such as congenital heart defects, can be further categorized. However, due to data limitations, we were unable to do it. Third, our study did not include birth defects before 28 weeks of gestation and after the first 42 days of life. However, as discussed above, many birth defects were diagnosed and terminated before 28 weeks of gestation (such as Down syndrome), and many birth defects may die after the first 42 days of life. Fourth, many cases had multiple defects, which could potentially affect the death rates. However, we did not analyze the combined effects.

## Conclusion

In summary, we have described the prevalence and epidemiology of birth defects from population-based surveillance in Hunan Province, China, 2010–2020. There were differences in the prevalence and death rate of birth defects between population-based surveillance and hospital-based surveillance.

## Data Availability

All data generated or analyzed during this study are included in this published article.
